# Counterfactual estimation of efficacy against placebo for novel PrEP agents using external trial data: example of injectable cabotegravir and oral PrEP in women

**DOI:** 10.1002/jia2.26118

**Published:** 2023-06-26

**Authors:** Deborah Donnell, Fei Gao, James P. Hughes, Brett Hanscom, Lawrence Corey, Myron S. Cohen, Srilatha Edupuganti, Nyaradzo Mgodi, Helen Rees, Jared M. Baeten, Glenda Gray, Linda‐Gail Bekker, Mina Hosseinipour, Sinead Delany‐Moretlwe

**Affiliations:** ^1^ Fred Hutchinson Cancer Center Seattle Washington USA; ^2^ University of Washington Seattle Washington USA; ^3^ University of North Carolina Chapel Hill North Carolina USA; ^4^ Emory University Atlanta Georgia USA; ^5^ University of Zimbabwe Clinical Trials Research Centre Harare Zimbabwe; ^6^ Wits RHI Johannesberg South Africa; ^7^ South Africa Medical Research Council Tygerberg South Africa; ^8^ University of Cape Town Cape Town South Africa

**Keywords:** counterfactual placebo, cabotegravir, emtricitabine/tenofovir disoproxil fumarate, HIV, pre‐exposure prophylaxis, efficacy trial design

## Abstract

**Introduction:**

Multiple antiretroviral agents have demonstrated efficacy for human immunodeficiency virus (HIV) pre‐exposure prophylaxis (PrEP). As a result, clinical trials of novel agents have transitioned from placebo‐ to active‐controlled designs; however, active‐controlled trials do not provide an estimate of efficacy versus no use of PrEP. Counterfactual placebo comparisons using other data sources could be employed to provide this information.

**Methods:**

We compared the active‐controlled study (HPTN 084) of injectable cabotegravir (CAB‐LA) versus daily oral emtricitabine/tenofovir disoproxil fumarate (FTC/TDF) among women from seven countries in Africa to three external, contemporaneous randomized HIV prevention trials from which we constructed counterfactual placebo estimates. We used direct standardization via analysis weights to achieve the same distribution of person‐years between the external study and HPTN 084, across strata predictive of HIV risk (country and selected risk covariates). We estimated prevention efficacy against a counterfactual placebo to provide information on the use of CAB‐LA and FTC/TDF compared to no intervention. We compared the counterfactual placebo findings for FTC/TDF to previous placebo‐controlled trials, adjusted for observed adherence to daily pills.

**Results:**

Distribution of age and baseline prevalence of gonorrhoea and chlamydia were similar among matched counterfactual placebo and observed HPTN 084 arms after standardization. Counterfactual estimates of CAB‐LA versus placebo in all three settings showed a consistent risk reduction of 93%–94%, with lower bounds of the confidence intervals above 72%. Observed adherence (quantifiable tenofovir in plasma) in HPTN 084 was 54%–56%, and estimated efficacy of daily oral FTC/TDF against a counterfactual placebo was consistent with a predicted risk reduction of 39%–40% for this level of daily pill use.

**Conclusions:**

Counterfactual placebo rates of HIV acquisition derived from external trial data in similar locations and time can be used to support estimates of placebo‐based efficacy of a novel HIV prevention agent. External trial data must be standardized to be representative of the clinical trial cohort testing the novel HIV prevention agent, accounting for confounders.

## 1 INTRODUCTION

The daily oral pill pre‐exposure prophylaxis (PrEP) regimen containing emtricitabine/tenofovir disoproxil fumarate (FTC/TDF) reduces human immunodeficiency virus (HIV) by >90%, when used as prescribed [1, 2, 3]. While “biological efficacy” of FTC/TDF is high, the degree of protection achieved is diminished by non‐adherence [4, 5, 6, 7, 8]. Unfortunately, adherence to daily FTC/TDF is challenging in both placebo‐controlled studies of women and PrEP demonstration projects in women, where adherence rates were low and discontinuation rates after 6 months were high [4].

Adherence challenges galvanized the development of new HIV prevention products with trial designs that used daily oral FTC/TDF as an active comparator [9, 10, 11, 12]. For example, two double‐blind double‐dummy active‐control randomized clinical trials (RCTs) of injectable cabotegravir (CAB‐LA) every 2 months compared to daily oral FTC/TDF were conducted (HPTN 083 and HPTN 084); both trials resulted in substantially lower risk of HIV acquisition in the CAB‐LA compared to FTC/TDF arm. These active‐control designs estimated prevention efficacy compared to a proven active agent (i.e. oral FTC/TDF). However, clinicians and patients often want to know how well something works against placebo (i.e. absolute effectiveness) [13], not how well it works relative to an alternative intervention. In contraception, for example, multiple options exist, and patient discussions are framed by relative pregnancy prevention of each against a hypothetical placebo (i.e. nothing). Similarly, estimates of the efficacy for CAB‐LA compared to a hypothetical placebo will likely be important for decision‐making about HIV prevention.

After clinically meaningful prevention efficacy and safety is established in an RCT with a placebo arm, subsequent randomized efficacy trials compare two active agents (novel vs. proven) in an active‐control trial, typically using a non‐inferiority design [14]. A challenge in HIV prevention is that fully powered non‐inferiority designs of active‐controlled trials will not be feasible when the risk of infection is reduced to rates well below 1/100 person‐years (PYs) through the use of prevention agents with proven efficacy >90%. Thus, alternative strategies for evaluating the efficacy of novel products are needed. One potential method is to construct a synthetic placebo comparison by leveraging pre‐existing or concurrent placebo data from external trials [15].

Here, we demonstrate the use of counterfactual placebo methodology to estimate HIV prevention efficacy compared to placebo for participants enrolled in the active‐controlled HPTN 084 study, using HIV diagnosis outcome data from three concurrent trials that prospectively measured HIV incidence in women assigned to placebo or products that were not HIV prevention agents. Using HIV acquisition rates observed in these three external trials, we estimate the placebo‐based efficacy of CAB‐LA. We also estimate the efficacy of oral FTC/TDF, the other active prevention arm of the HPTN 084 study, using a counterfactual placebo methodology. This is then compared to FTC/TDF efficacy for the observed adherence to daily pills estimated from prior RCTs as a validation of the methodology.

## 2 METHODS

The active controlled study, HPTN 084, was a double‐blinded RCT of injectable CAB‐LA versus oral FTC/TDF in 3224 women from Botswana, Eswatini, Kenya, Malawi, South Africa and Zimbabwe. Participants received HIV testing to identify new diagnoses and assess incidence rates for each product using an intent‐to‐treat approach, that is including all follow‐ups irrespective of product use. A group of 400 women from the FTC/TDF arm were randomly selected for longitudinal biomarker testing via plasma and dried blood spot (DBS) samples to assess adherence. Tenofovir concentrations were measured in plasma and tenofovir‐diphosphate (TFV‐DP) in DBS samples [16, 17].

Enrolment was initiated in November 2017, and the study was unblinded early for demonstrated superiority in November 2020. The trial found a risk reduction of 88% (95% confidence interval [CI]: 69%–95%) for CAB‐LA compared to FTC/TDF. Four new diagnoses were observed in women randomized to CAB‐LA (incidence = 0.20/100 person years), compared to 36 in the FTC/TDF arm (incidence = 1.85/100 PY) [12]. Our study includes all newly diagnosed HIV observed up to the study unblinding.

Data from three external, concurrent trials that enrolled women in African countries provide HIV incidence rates for women not using prevention products:

1. The Antibody Mediated Prevention trial (AMP‐women's: HVTN703/HPTN 081) randomized women aged 18−40 years to 1:1:1 to blinded infusion of a placebo, low, or high dose of the monoclonal antibody VRC01 [18, 19]. The AMP women's trial enrolled 1924 women in seven countries in southern Africa (Botswana, Kenya, Malawi, Mozambique, South Africa, Tanzania and Zimbabwe) between May 2016 and September 2018. Women were tested for HIV every 4 weeks. Our study includes all new diagnoses observed on the placebo arm up through the end of 104 weeks of follow‐up.
2. The HVTN 702 RCT enrolled 3786 women in South Africa aged 18–35 and randomized them 1:1 to ALVAC‐HIV and bivalent Subtype C gp120‐MF59 versus placebo [20]. Participants were tested for HIV every 3 months. The trial recruited between October 2016 and June 2019; in January 2020, the vaccinations were halted for lack of efficacy. This analysis includes all new diagnoses observed among women on the placebo arm through February 2020.
3. The Evidence for Contraceptive Options in HIV Outcomes (ECHO) trial enrolled 7829 women aged 18–35 in four countries (Eswatini, Kenya, South Africa and Zambia) and randomized them 1:1:1 to either DMPA‐IM, levonorgestrel implant or copper intrauterine device [21]. Accrual occurred between December 2015 and September 2017, with follow‐up visits through October 2018. Women were followed for a maximum of 18 months, and received an HIV test every 3 months. The study products randomized in ECHO were not HIV prevention agents, so we included the entire cohort in the counterfactual placebo analyses.

**Figure 1:**
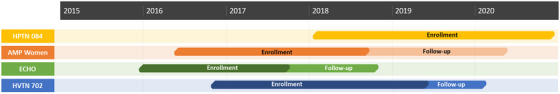
The timeline and duration (time from first enrolment to last visit included in analysis) are shown for the four studies. Enrolment period is indicated in darker hue, period of continued follow‐up of enrolled participants in lighter hue.

Trial inclusion criteria were similar (see Table S1). At enrolment, participants were included if they were without HIV at screening, were sexually active and considered behaviourally vulnerable for HIV, non‐pregnant and willing to use effective contraception if capable of becoming pregnant, generally in good health, able to consent and willing to comply with trial procedures. ECHO excluded women not capable of becoming pregnant. See Tables S2 and S3 for trial sites, participants, person‐years and dates of conduct. Follow‐up time was censored at the time a woman was first diagnosed with HIV. See Figure 1 for the overlap in recruitment and follow‐up time of the studies.

All four trials included regular HIV counselling and testing, provision of condoms, sexual behaviour and pregnancy prevention counselling, and syndromic screening or testing for two sexually transmitted infections (STIs), *Neisseria gonorrhoea* and *Chlamydia trachomatis*, with treatment per local standard of care. In the three external trials used in the counterfactual placebo analyses, oral FTC/TDF PrEP was available, primarily by referral governed by local practise; however, PrEP uptake was low: TFV‐DP was quantified in DBS in 3.8% in the AMP‐women's study [19] and in 2% of participants in HVTN 702; [20] 8% of women initiated PrEP in ECHO, almost exclusively in the last months of the trial [22].

In HVTN 702 and AMP‐women's trial, DBS from both active and placebo arms were collected and tested for TFV‐DP in a random subset of visits. Benchmark studies of tenofovir biomarkers of adherence established that in plasma, tenofovir remains quantifiable for 2 weeks, in DBS, TFV‐DP remains quantifiable for 3 months and TFV‐DP >700 fmol/punch is consistent with taking four or more pills per week [16, 17].

In all trials, individual informed consent processes were maintained, community advisory boards and other stakeholders engaged, and local ethics boards provided oversight. Trials were registered on ClinicalTrials.gov (HPTN 084: NCT03164564; HVTN 703/HPTN 081: NCT02716675; HVTN 702: NCT02968849; ECHO: NCT02550067).

### 2.1 Statistical methods

Counterfactual comparisons were computed for HPTN 084 using each external study placebo group. To ensure individuals in HPTN 084 and the external study groups were comparable, each comparison was restricted to women in the same age range and countries participating in both HPTN 084 and the external study (aligning with the “positivity assumption” of causal inference) [23]. We defined counterfactual placebo comparisons based on three studies in three geographic settings: (1) five‐country setting, using data from the AMP‐women's study, with representation from Botswana, Kenya, Malawi, South Africa and Zimbabwe; (2) three‐country setting based on the ECHO study sites in Eswatini, Kenya and South Africa; and (3) comparisons from South Africa only, based separately on the ECHO and HVTN 702 studies. For multi‐country comparisons, country was used as a standardization strata, because country was strongly predictive of HIV incidence rate. Our approach constructs a multi‐site placebo arm from mutually participating countries to induce a close match in HIV likelihood characteristics between included HPTN 084 participants and their counterfactual placebo comparison. The South Africa comparison used age categories as a standardization strata. Lastly, we also stratified on STI at baseline diagnosis of gonorrhoea or chlamydia, to account for sexual behaviour. Baseline demographics were compared for participants in HPTN 084 against external studies (country‐ or age‐standardized), approximating the comparison of characteristics in an RCT arm (see Table S4).

Counterfactual HIV incidence rates were constructed based on matching the person‐year distribution of HPTN 084 by direct standardization of country‐STI strata. For each external study, counterfactual placebo HIV incidence is estimated by $\sum_{i}w_{i}I_{i}$, where $I_{i}$ is the observed placebo/no treatment‐arm incidence in the *i*th country‐STI strata in the external study and $w_{i}=\frac{m_{i}}{\sum_{i}m_{i}}\;\;$is the incidence analysis weight ($m_{i}$ is the person years in the *i* ^*th*^ country‐STI strata for HPTN 084). As detailed in the Supplement, this is equivalent to applying individual analysis weight $w_{ij}^{*}=\frac{m_{i}}{n_{i}}\;\frac{\sum_{i}n_{i}}{\sum_{i}m_{i}}$ to each participant in the *i*th country‐STI strata in the external study, where $n_{i}$ is the person years in the *i*th country‐STI strata for the external study. Estimates and standard errors of the log incidence rate are calculated assuming a Poisson distribution for HIV incidence on the individual‐level data in the external studies with sampling weights (see Supplement). Comparisons limited to South Africa only were age‐STI‐standardized, where weights and incidences were calculated by age group (18–20, 21–25, 26–30 and 31–35) and STI using the same formula, indexing age group instead of country.

Efficacy was estimated as one minus the ratio of HPTN 084 arm‐specific and counterfactual incidence. Variance (on the log incidence scale) was estimated assuming independence between studies and incorporated the use of weights (see Supplement). The counterfactual efficacy estimate assumes that incidence rates from the external studies are representative of the HPTN 084 cohort within each country‐/age‐strata in the absence of study provision of active PrEP product. Analysis was done in R, version 4.0.3, using package *survey*.

Because the efficacy of FTC/TDF versus placebo is well characterized from multiple RCTs, we compared the counterfactual‐based placebo efficacy to the predicted risk reduction, given adherence to FTC/TDF in HPTN 084, based on prior results. Specifically, the efficacy of FTC/TDF is predicted based on observed adherence (i.e. proportion of visits with quantifiable plasma) using the published meta‐regression relationship of plasma‐based adherence and efficacy in men and women: [24, 25] the relative risk of FTC/TDF (A) versus placebo (P) in women is:

$$RR_{\frac{A}{P}}\left(p_{adh}\right)=\;exp\left(0.7520\;-2.276\times \;p_{adh}\right),$$

where $p_{adh}$ is the proportion of FTC/TDF arm participants with quantifiable tenofovir concentrations in plasma. Observed adherence is calculated for each setting (five‐country, three‐country and South Africa) to predict efficacy for that comparison. FTC/TDF was available to all participants in the external studies per local standard of care, and we report the observed use of FTC/TDF for the counterfactual placebo for AMP women and HVTN 702, as they evaluated biomarkers of PrEP use.

## 3 RESULTS

All three settings included a preponderance of women aged 18–25 in HPTN 084: 54.9% in the five‐country comparison, 68.7% in the three‐country and 72.5% in South Africa (Table 1). Baseline prevalence of gonorrhoea (6.9%–7.6%) and chlamydia (20.8%–25.6%) in HPTN 084 were similar across settings. Note that our standardization matched the person‐year distribution across strata defined by country and STIs between HPTN 084 for AMP‐women and ECHO, without explicit adjustment for age, and the distribution of age for each was similar to HPTN 084.

**Table 1:** Comparison of baseline characteristics of women in the active‐control study, HPTN 084 and three external studies.

		Five‐country setting (AMP‐women placebo, age 18–40)			Three‐country setting (ECHO, age 18–35)			South Africa only setting (HVTN 702 placebo, ECHO, age 18–35)			
Variable		HPTN 084 CAB‐LA (*N* = 1206)	HPTN 084 FTC/TDF (*N* = 1205)	Weighted AMP‐women (*N* = 616)	HPTN 084 CAB‐LA (*N* = 728)	HPTN 084 FTC/TDF (*N* = 741)	Weighted ECHO (*N* = 7104)	HPTN 084 CAB‐LA (*N* = 624)	HPTN 084 FTC/TDF (*N* = 632)	Weighted HVTN 702 (*N* = 1886)	Weighted ECHO (*N* = 5712)
Age: mean (sd)		26.02 (5.37)	26.17 (5.36)	26.87 (5.13)	24.20 (4.30)	24.05 (3.91)	23.67 (4.18)	23.84 (4.18)	23.80 (3.86)	23.87 (3.97)	23.81 (4.06)
Age category	18–20	14.4%	13.6%	12.0%	20.5%	19.3%	26.7%	22.5%	21.1%	21.7%	21.7%
	21–25	40.5%	39.9%	33.4%	48.2%	49.2%	43.6%	50.0%	50.2%	50.4%	50.4%
	26–30	23.4%	24.2%	29.5%	19.1%	23.1%	21.0%	17.0%	20.6%	18.7%	18.7%
	31–35	14.2%	15.5%	20.1%	12.3%	8.4%	8.6%	10.5%	8.2%	9.2%	9.2%
	35–40	7.5%	6.8%	5.1%	−	−	−	−	−	−	−
Baseline gonorrhoea^a^		7.1%	6.4%	6.5%	6.9%	7.1%	6.9%	7.6%	7.8%	7.6%	7.6%
Baseline chlamydia^a^		20.8%	18.7%	19.7%	25.2%	22.3%	23.8%	25.6%	24.3%	24.8%	24.8%

Note: Women were selected for inclusion from each study based on overlapping site of enrolment and age inclusion criteria for HPTN084 and the external study. Characteristics are compared standardized by person years contributed by strata (see Supplement for details).

Abbreviations: CAB‐LA, injectable cabotegravir; FTC/TDF, emtricitabine/tenofovir disoproxil fumarate.

^a^ Baseline STI evaluated any positive NAAT test on a collected sample. HPTN 084 STI samples included urine or vaginal swabs, AMP‐women's (HVTN704/HPTN 081) and HVTN 703 (vaccine) included urine, vaginal and cervical specimens, ECHO included endocervical swabs. STI testing was missing on 13% of women in HVTN 702 because STI testing was initiated in version 2 of the protocol.

### 3.1 Efficacy of CAB‐LA compared to counterfactual placebo

The five‐country counterfactual efficacy estimate of CAB‐LA from the AMP‐women's study was 92.8% (95% CI: 76.1%–97.8%), with a counterfactual placebo incidence of 2.78/100 PY (Table 2). This included 96% (1106 PY) of the follow‐up time in the AMP‐women's study and 77% (1498 PY) in the HPTN 084 study. The counterfactual efficacy for the three‐country ECHO‐based comparison was 94.7% (95% CI: 78.8%–98.7%), with a counterfactual placebo incidence of 4.74/100 PY based on 9425 PY. The estimated efficacy of CAB‐LA in South Africa based on 2335 PY from placebo‐arm women in the HVTN 702 study was 93.3% (95% CI: 72.8%–98.3%); and based on 7571 PY of ECHO study follow‐up from South Africa was 94.0% (95% CI: 76.0%–98.5%). Counterfactual placebo incidence in South Africa was 4.42/100 PY for HVTN 702 and 4.95/100 PY for ECHO, as compared with 0.30/100 PY for CAB‐LA participants in HPTN 084.

**Table 2:** Counterfactual placebo efficacy for CAB‐LA and FTC/TDF.

	Included PY/total study PY (%)			HIV incidence			Efficacy: 1 – HR (95% CI)	
CF placebo study (*N* participants included from study)	CF placebo PY PY/total PY (%)	CAB‐LA PY PY/total PY (%)	FTC/TDF PY PY/total PY (%)	CF placebo incidence (standardized)	CAB‐LA incidence	FTC/TDF incidence	CAB‐LA efficacy versus CF placebo HR (95% CI)	FTC/TDF efficacy versus CF placebo HR (95% CI)
AMP‐women placebo arm, 18–40 (*N* = 619) (Five‐country setting)	1106/1150 (96%)	1498/1956 (77%)	1465/1942 (75%)	2.78	0.20	2.32	92.8 (76.1, 97.8)	16.5 (−40.2, 50.3)
ECHO, 18–35 (*N* = 7171) (Three‐country setting)	9425/10,456 (90%)	798/1956 (41%)	793/1942 (41%)	4.74	0.25	2.65	94.7 (78.8, 98.7)	44.2 (13.3, 64.0)
HVTN 702 women placebo arm, 18–35 (*N* = 1884) (South Africa only setting)	2335/2785 (84%)	675/1956 (35%)	675/1942 (35%)	4.42	0.30	3.11	93.3 (72.8, 98.3)	29.5 (−13.0, 56.1)
ECHO, 18–35 (*N* = 5768) (South Africa only setting)	7571/10,456 (72%)	675/1956 (35%)	675/1942 (35%)	4.95	0.30	3.11	94.0 (76.0, 98.5)	37.2 (2.2, 59.6)

Note: Women were selected for inclusion for each comparison based on overlapping site of enrolment and age inclusion criteria for HPTN 084 and the external study.

Abbreviations: CAB‐LA, cabotegravir; CF, counterfactual; CI, confidence interval; FTC/TDF, emtricitabine/tenofovir disoproxil fumarate; HR, hazard ratio; PY, person‐years.

### 3.2 Efficacy of FTC/TDF compared to counterfactual placebo

The estimated proportion of visits with quantifiable plasma tenofovir in the FTC/TDF arm of HPTN 084, reflecting any use in the last 2 weeks, was 55.9% for the five‐country setting, 54.3% for the three‐country setting and 54.8% for South Africa (Table 3). TFV‐DP in DBS >700 fmol/punch, reflecting consistent adherence, was substantially smaller, ranging from 20.1% to 21.0% of participants (see Table S6). Using the same counterfactual placebo comparison data from external studies, the estimate for efficacy of FTC/TDF (as taken in HPTN 084) in the five‐country setting was 16.5% (40.2%, 50.3%), and in the three‐country setting was 44.2% (95% CI: 13.3%–64.0%). The estimated efficacy of FTC/TDF in South Africa based on HVTN 702 was 29.5% (95% CI: 13.0%–56.1%) and 37.2% (95% CI: 2.2%–59.6%) based on ECHO (Table 2).

**Table 3:** Biomarkers of adherence to FTC/TDF in HPTN 084 FTC/TDF arm, and corresponding efficacy based on meta‐regression model using evidence from prior placebo randomized trials.

Counterfactual group	HPTN 084 FTC/TDF arm: proportion with quantifiable tenofovir in plasma	Estimated efficacy based on adherence meta‐regression model
AMP women, age 18–40 (Five‐country setting)	55.9%	40.6% (22.2%, 54.6%)
ECHO, age 18–35 (Three‐country setting)	54.3%	38.4% (20.0%, 52.5%)
HVTN 702 and ECHO, age 18–35 (South Africa only setting)	54.8%	39.1% (20.7%, 53.2%)

To illustrate the sensitivity of the results to potential confounding, the analyses were repeated for efficacy for both CAB‐LA and TDF/FTC, using the same data but without stratification for STI at baseline, where STI is theorized to potentially affect both likelihood of HIV and use of PrEP. This results in a slight imbalance in the STIs at baseline, with lower STI rates observed in the external studies (Table S4). Not stratifying for STIs did not change the high efficacy estimates of CAB‐LA, but resulted in somewhat conservative efficacy estimates (up to 6% lower) for FTC/TDF (see Table S5).

The meta‐regression model for efficacy predicted for observed quantifiable plasma tenofovir based on prior RCTs estimated HIV risk reduction in HPTN 084 ranging from 40.6% to 38.4% across the three settings (Table 3).

## 4 DISCUSSION

Using counterfactual placebo methodology, CAB‐LA showed an estimated risk reduction of 92.8%–94.7% for women. HPTN 084 previously reported CAB‐LA's intent‐to‐treat efficacy as 88% relative to daily oral FTC/TDF [12]. Together, these findings support the high prevention efficacy of this long‐acting injectable in women compared to FTC/TDF and compared to a placebo. The counterfactual placebo methodology employed here provides an estimate that can be used when discussing how well CAB‐LA works compared to no intervention, information not obtainable from the active‐control study.

Recent regulatory guidance on the use of external controls, while appropriately cautious, acknowledges the greater potential for the reliability of external data derived from clinical trials [26]. Our counterfactual placebo methodology relies on the assumption that strata‐standardized rates of HIV diagnosis without PrEP from the external studies are comparable to an HPTN 084 placebo arm. Because our placebo counterfactuals were based on prospective follow‐up in HIV prevention trials conducted at similar geographic locations and times, and because two of the trials had placebo arms, they closely approximate HIV incidence for participants taking no active agent in an HIV prevention trial. The balance for age between the external studies and HPTN 084 arms for each comparison offers important support for this assumption.

An asset of counterfactual placebo methodology is that findings are not in comparison to an active control. For example, with the active‐controlled trial used for this analysis (HPTN 084), the expected efficacy of oral FTC/TDF depended on adherence, therefore, the relative efficacy of CAB‐LA decreases as adherence to FTC/TDF increases. Instead, our placebo counterfactuals leveraged the prevention trial landscape of 2015–2020, where RCTs of non‐PrEP products (e.g. vaccines and monoclonal antibodies) were operating at the same regions and time as the active control study, HPTN 084. A notable strength of our study is the availability of individual‐level, contemporary HIV incidence data from multiple high‐quality RCTs. Specifically, they came from three settings, simulating estimates from multi‐site trials conducted in different countries. Analogous to finding similar efficacy in trials conducted in different settings, the consistency of our estimates across multiple settings from multiple external studies, and placebo incidence ranging from 2.78 to 4.95/100 PY, increases confidence in these counterfactual placebo findings and methodology. This also allays concerns inherent to typical historical comparison data, where secular changes in the epidemic (i.e. decreased HIV incidence resulting from increased PrEP and HIV testing) or passive surveillance/routine monitoring data with less intensive follow‐up processes, can lead to comparisons that are more likely to have important confounders. That said, concurrently running placebo‐controlled trials is unlikely in future HIV prevention trials, so this current counterfactual analysis capitalized on a unique circumstance. Future HIV prevention trials are likely to depend on historical placebo incidence data, and additional work is needed to rigorously examine sensitivity to the additional assumptions required when using historical data. Adequate control of confounding is also critical [27], as illustrated by the changes in our FTC/TDF estimates as a result of not stratifying for STIs; assessing sensitivity to potential bias in placebo counterfactual incidence rates is also important.

The counterfactual placebo estimates of oral FTC/TDF efficacy ranged from 15% to 40%, in the context of modest adherence to oral PrEP in HPTN 084: 45% of women were not taking FTC/TDF regularly (with quantifiable tenofovir in 55% of samples). Given this level of adherence, evidence from previous placebo‐controlled RCTs predicted that FTC/TDF would reduce the risk of HIV about 40% [24, 25]. Our counterfactual placebo efficacy estimates from ECHO and HVTN 702 closely matched this reduction, and were within the confidence limits for the AMP‐women's study (notably the external study with the smallest number of person‐years). The higher variability in these estimates, together with the observed sensitivity to adjustment for baseline STI are cautionary for using external counterfactuals where adherence is difficult to predict.

When a counterfactual placebo is used to estimate efficacy, just as for any RCT, power to detect an effect is a function of the relative efficacy of the interventions. With CAB‐LA—an intervention with high (>90%) efficacy, counterfactual efficacy estimates show consistent and unequivocal efficacy compared to no PrEP use. However, for FTC/TDF, two of the four counterfactual efficacy estimates included 0, that is did not rule out lack of efficacy. Notably, the comparisons excluding zero were based on ECHO, the trial with the largest person‐years. Thus, planning a well‐powered counterfactual placebo efficacy estimate requires a highly efficacious intervention, or sufficiently large person‐years in both experimental and placebo follow‐up to achieve a reliable estimate of a modest effect.

Our method used direct standardization (with the clinical trial of the experimental product as the reference population) of placebo data from external studies, which resulted in the high similarity between trial populations after standardization. There is a rich literature of methodology for the estimation of direct causal effects using counterfactuals in the context of both observational and randomized studies [28, 29, 30], which differ from the intent‐to‐treat estimand of our direct standardization approach. Mathematical models also use counterfactual scenarios to model the impact of new interventions based on sets of complex assumptions informed by clinical trial results [31, 32, 33, 34]. Our analysis presages a future approach to estimating the efficacy of new prevention products in specific high‐incidence populations, based on the use of placebo‐incidence data from comparable clinical trial settings. Importantly, the rigour of clinical trial evaluation largely mitigates the concern of different quality of ascertainment in comparisons based on external studies. This complements other approaches to bridging from placebo data in the same high‐incidence populations [35, 36, 37, 38].

Limitations of our counterfactual approach correspond to those of any non‐randomized comparison: potential for bias due to confounding (e.g. sexual behaviour). Women in the four included trials were recruited separately, and our comparisons were not protected by randomization, that is differences could be attributable in part to differences in study populations. The ECHO Trial enrolled women interested in pregnancy prevention, not HIV prevention, yet inclusion and exclusion criteria were similar to the other trials and participants were aware that the purpose of the trial was to investigate contraception in association with the likelihood of acquiring HIV. A further limitation was the inability to explicitly match or report on sexual behaviour, as comparable measures were not collected; however, the adjustment for baseline STI likely largely achieved balance. While all three external study settings included the potential for access to oral FTC/TDF, uptake was low during study follow‐up, thus PrEP would not have substantially decreased HIV incidence in the placebo counterfactual.

## 5 CONCLUSIONS

Counterfactual placebo estimates, based on data from external studies matched in time and location, generated placebo‐controlled estimates of efficacy in the absence of a randomized placebo group. Our counterfactual placebo method provides a useful proof‐of‐concept for planning future intervention trials that use active control or have high levels of prevention product use in the standard of prevention. Counterfactual placebo rates of HIV acquisition derived from external trial data in similar locations and time can be used to support estimates of placebo‐based efficacy of a novel HIV prevention agent. External trial data must be standardized to be representative of the clinical trial cohort testing the novel HIV prevention agent, accounting for confounders.

## COMPETING INTERESTS

The authors report no competing interests.

## AUTHORS’ CONTRIBUTIONS

DD and FG designed the study, performed the analysis and wrote the paper. LC, MSC, SE and NM were lead scientists of the HVTN703/HPTN 081 study. HR, JMB and DD were lead scientists of the ECHO study. GG, L‐GB and LC were lead scientists of the HVTN702 study. SD‐M, MH, JPH and BH were lead scientists of the HPTN 084 study. Each team provided data access and approved the study design. All authors reviewed and provided critical feedback on the manuscript.

## FUNDING

This work was made possible through funding support from the National Institute of Allergy and Infectious Diseases, Office of the Director, National Institutes of Health (NIH), National Institute on Drug Abuse and the National Institute of Mental Health, under award numbers UM1AI068619 (HIV Prevention Trials Network [HPTN] Leadership and Operations Center), UM1AI068617 (HPTN Statistical and Data Management Center [SDMC]), UM1AI068613 (HPTN Laboratory Center), UM1 AI068614 (HIV Vaccine Trials Network [HVTN] Leadership and Operations Center), UM1 AI068635 (HVTN SDMC) and UM1 AI068618, UM1 AI068619 (HVTN Laboratory Center). ECHO was funded by Bill & Melinda Gates Foundation, US Agency for International Development and the President's Emergency Plan for AIDS Relief, Swedish International Development Cooperation Agency, South African Medical Research Council and UN Population Fund. Additional funding was provided to HPTN 084 by the Bill & Melinda Gates Foundation (OPP1154174) and ViiV Healthcare. Pharmaceutical support was provided by ViiV Healthcare and Gilead Sciences.

## DISCLAIMER

The content is solely the responsibility of the authors and does not necessarily represent the official views of the NIH.

## Supporting information

Supporting Information

## Data Availability

Data from the ECHO trial, HVTN702 trial and AMP women's (HVTN/HPTN 081) trial are available. Data from HPTN 084 trial are available from dataaccess@scharp.org.
