# Supplementary material for: Counterfactual estimation of efficacy against placebo for novel PrEP agents using external trial data: example of injectable cabotegravir and oral PrEP in women
Source: J Int AIDS Soc. 2023 Jun 26;26(6):e26118. doi: 10.1002/jia2.26118 (PMC10292682; doi:10.1002/jia2.26118)
Supplement: Supplementary file 1 — Supporting Information [file JIA2-26-e26118-s001.docx]

**Supplement to Counterfactual Estimation of Efficacy against Placebo for novel PrEP Agents using External Trial Data: example of injectable Cabotegravir and oral PrEP in women**

**Table of Contents**

[**Table S1: Comparison of major inclusion and exclusion criteria for cisgender women in the trials** 2](#_Toc132300263)

[**Table S2. Number of sites, participants, and person years by country for complete follow-up from women in HPTN 084 and each external study** 3](#_Toc132300264)

[**Table S3. Dates of trial conduct** 4](#_Toc132300265)

[**Table S4.** **Comparison of baseline characteristics of women in the active control study, HPTN 084, and three external studies, without stratification for STI.** Women were selected for inclusion from each study based on overlapping site of enrolment and age inclusion criteria for HPTN084 and the external study. Characteristics are compared standardized by person years contributed by strata of Country (Age for South Africa) only (i.e. without STI strata) 5](#_Toc132300266)

[**Table S5. Counterfactual placebo efficacy for CAB-LA and FTC/TDF standardized by country or age and additionally for STI** 6](#_Toc132300267)

[**Table S6. DBS Biomarker measuring PrEP use in HPTN 084, AMP-women’s and HVTN702 trials** 7](#_Toc132300268)

[**Methods: Analysis method for counterfactual incidence estimation** 8](#_Toc132300269)

[**R code for the counterfactual analysis** 9](#_Toc132300270)

# **Table S1: Comparison of major inclusion and exclusion criteria for cisgender women in the trials**

|  | **HPTN 084** | **HVTN702** | **ECHO** | **AMP women** |
| --- | --- | --- | --- | --- |
| Age | 18-45 years | 18 to 35 years | 18-35 years | 18 to 40 years |
| HIV at baseline | Non-reactive HIV test results at Screening and Enrollment | Negative HIV-1 and -2 blood test within 30 days prior to enrollment | HIV-seronegative | HIV uninfected, within 30 days prior to enrollment |
| Sexual risk | Sexually active (i.e., vaginal intercourse on a minimum of two separate days in the 30 days prior to Screening) and  Score of >5 using a modified VOICE risk score | Sexually active, defined as having had sexual intercourse at least twice in the past 30 days prior to screening, and considered by the site staff to be at risk for HIV infection | Is sexually active (has had vaginal sex within the last 3 months) or was pregnant within the last 3 months | In the 6 months prior to randomization, has had vaginal and/or anal intercourse with a male partner. Volunteers in a mutually monogamous relationship with an HIV-1 seronegative partner for > 1 year are excluded. |
| Contraception | Agree to use a reliable form of long-acting contraception, during the trial:   - Intrauterine device - Hormone-based contraceptive that meets <1% failure rate (implants or injectables only; this excludes combined oral contraception)   OR  Have documented evidence of surgical sterilization, or no uterus (e.g. hysterectomy) | Agree to consistently use effective contraception during the trial, defined as using 2 methods of birth control. These include 1 of the following methods:   - Condoms (male or female), Diaphragm or cervical cap   PLUS 1 of the following methods:   - Intrauterine device (IUD) or Hormonal contraception   OR  Not be of reproductive potential, such as menopause hysterectomy, bilateral oophorectomy, or tubal ligation. | Wants to use effective contraception, agrees to be randomised to either DMPA, NET-En, levonorgestrel implant, or copper IUD, and agrees to use assigned method for at least 18 months  Has not used DMPA, Net-En, a contraceptive implant, or an IUD in the last 6 months  Has not had a hysterectomy or sterilization | Agree to consistently use effective contraception  OR  Not capable of becoming pregnant due to having undergone total hysterectomy or bilateral oophorectomy (verified by medical records) |
| Pregnancy | Not Pregnant or currently breastfeeding, or intends to become pregnant and/or breastfeed during the study | Not Pregnant or breastfeeding | Not pregnant or intending to become pregnant in the next 18 months | Not Pregnant or breastfeeding |
| Weight | No restriction | No restriction | No restriction | Body mass index (BMI) < 40 |

Table does not include medical eligibility criteria specific to study products (e.g., known allergies, drug or pre-existing conditions that are counterindications to participation)

# **Table S2. Number of sites, participants, and person years by country for complete follow-up from women in HPTN 084 and each external study**

| **Trial** | **HPTN 084** | | | | | **AMP Women’s Placebo** | | | **ECHO** | | | **HVTN 702 Placebo** | | |
| --- | --- | --- | --- | --- | --- | --- | --- | --- | --- | --- | --- | --- | --- | --- |
| **Country** | **# Sites** | **Cabotegravir** | | **TDF/FTC** | | **# Sites** | **# Sub** | **PY** | **# Sites** | **# Sub** | **PY** | **# Sites** | **# Sub** | **PY** |
|  |  | **# Sub** | **PY** | **# Sub** | **PY** |  |  |  |  |  |  |  |  |  |
| Botswana | 1 | 46 | 69.4 | 45 | 68.8 |  |  |  |  |  |  |  |  |  |
| Eswatini | 1 | 80 | 82.7 | 80 | 83.6 | 1 | 47 | 97.3 | 1 | 502 | 663.7 |  |  |  |
| Kenya | 1 | 31 | 50.4 | 35 | 53.2 |  |  |  | 1 | 901 | 1250.4 |  |  |  |
| Malawi | 2 | 113 | 144.9 | 111 | 133.4 | 1 | 27 | 52.3 |  |  |  |  |  |  |
| Mozambique |  |  |  |  |  | 2 | 59 | 110.1 |  |  |  |  |  |  |
| South Africa | 7 | 653 | 722.7 | 655 | 719.1 | 1 | 8 | 13.1 | 9 | 5768 | 7679.6 | 14 | 1884 | 2782.2 |
| Tanzania |  |  |  |  |  | 6 | 341 | 637.2 |  |  |  |  |  |  |
| Uganda | 3 | 300 | 326.4 | 296 | 327.8 | 1 | 11 | 20.2 |  |  |  |  |  |  |
| Zambia |  |  |  |  |  |  |  |  | 1 | 658 | 862.4 |  |  |  |
| Zimbabwe | 5 | 391 | 559.9 | 388 | 555.5 |  |  |  |  |  |  |  |  |  |
| Total | 20 | 1614 | 1956.3 | 1610 | 1941.6 | 3 | 145 | 272.3 | 12 | 7829 | 10456.1 | 14 | 1884 | 2782.2 |

# **Table S3. Dates of trial conduct**

|  | **Start accrual** | **Finish accrual** | **Finish date of follow-up** |
| --- | --- | --- | --- |
| HPTN 084 | Nov 27, 2017 | Nov 5, 2020 | Nov 5, 2020 |
| AMP Women’s | May 17 2016 | Sept 20 2018 | Apr 3, 2020 |
| HVTN 702 | Oct 26 2016 | June 21 2019 | Feb 18 2020 |
| ECHO | Dec 14 2015 | Sep 12 2017 | Oct 31, 2018 |

# **Table S4.** **Comparison of baseline characteristics of women in the active control study, HPTN 084, and three external studies, without stratification for STI.** Women were selected for inclusion from each study based on overlapping site of enrolment and age inclusion criteria for HPTN084 and the external study. Characteristics are compared standardized by person years contributed by strata of Country (Age for South Africa) only (i.e. without STI strata)

|  | | **Five-country**  (AMP-women Placebo, age 18-40) | | | **Three-country**  (ECHO, age 18-35) | | | **South Africa**  (HVTN 702 placebo, ECHO, age 18-35) | | | |
| --- | --- | --- | --- | --- | --- | --- | --- | --- | --- | --- | --- |
| **Variable** | | HPTN 084 CAB-LA  (N = 1206) | HPTN 084 FTC/TDF  (N= 1205) | Weighted AMP-women  (N = 616) | HPTN 084 CAB-LA  (N = 728) | HPTN 084 FTC/TDF  (N = 741) | Weighted ECHO  (N = 7104) | HPTN 084 CAB-LA  (N = 624) | HPTN 084 FTC/TDF  (N = 632) | Weighted HVTN 702  (N = 1886) | Weighted ECHO  (N = 5712) |
| Age: mean  (sd) | | 26.02  (5.37) | 26.17  (5.36) | 26.81  (5.12) | 24.20  (4.30) | 24.05  (3.91) | 23.74  (4.20) | 23.84  (4.18) | 23.80  (3.86) | 23.89  (4.00) | 23.83  (4.08) |
| Age Category | 18-20 | 14.4% | 13.6% | 11.2% | 20.5% | 19.3% | 26.3% | 22.5% | 21.1% | 21.8% | 21.8% |
|  | 21-25 | 40.5% | 39.9% | 33.7% | 48.2% | 49.2% | 43.4% | 50.0% | 50.2% | 50.1% | 50.1% |
|  | 26-30 | 23.4% | 24.2% | 29.8% | 19.1% | 23.1% | 21.5% | 17.0% | 20.6% | 18.8% | 18.8% |
|  | 31-35 | 14.2% | 15.5% | 20.1% | 12.3% | 8.4% | 8.9% | 10.5% | 8.2% | 9.3% | 9.3% |
|  | 35-40 | 7.5% | 6.8% | 5.2% | - | - | - | - | - | - | - |
| Baseline Gonorrhoea (%)† | | 7.1% | 6.4% | 5.1% | 6.9% | 7.1% | 4.8% | 7.6% | 7.8% | 4.9% | 4.9% |
| Baseline Chlamydia (%)† | | 20.8% | 18.7% | 15.3% | 25.2% | 22.3% | 19.8% | 25.6% | 24.3% | 22.1% | 21.0% |

CAB-LA: injectable cabotegravir

FTC/TDF: emtricitabine/tenofovir disoproxil fumarate

†Baseline STI evaluated any positive NAAT test on a collected sample. HPTN 084 STI samples included urine or vaginal swabs, AMP-women’s (HVTN704/HPTN 081) and HVTN 703 (vaccine) included urine, vaginal and cervical specimens, ECHO included endocervical swabs. STI testing was missing on 13% of women in HVTN 702 because STI testing was initiated in version 2 of the protocol

# **Table S5. Counterfactual placebo efficacy for CAB-LA and FTC/TDF standardized by country or age and additionally for STI**

|  | **Estimates standardized for country or age** | | **Standardized additionally for STI** | |
| --- | --- | --- | --- | --- |
| **CF placebo study**  **(N participants included from study)** | **CAB-LA Efficacy**  vs CF Placebo^1^  HR (95% CI) | **FTC/TDF Efficacy**  vs CF Placebo^1^  HR (95% CI) | **CAB-LA Efficacy**  vs CF Placebo^1^  HR (95% CI) | **FTC/TDF Efficacy**  vs CF Placebo^1^  HR (95% CI) |
| AMP-women Placebo arm,  Age 18-40 (N = 619)  (Five-country setting) | 92.8 (76.3, 97.8) | 14.5 (-39.1, 47.5) | 92.8 (76.1, 97.8) | 16.5 (-40.2, 50.3) |
| ECHO, age 18-35  (N = 7171)  (Three-country setting) | 94.5 (77.8, 98.6) | 39.9 (7.6, 60.9) | 94.7 (78.8, 98.7) | 44.2 (13.3, 64.0) |
| HVTN 702 Women Placebo arm, age 18-35 (N=1884)  (South Africa only setting) | 93.0 (71.8, 98.3) | 24.2 (-19.6, 52.0) | 93.3 (72.8, 98.3) | 29.5 (-13.0, 56.1) |
| ECHO, age 18-35  (N = 5768)  (South Africa only setting) | 93.7 (74.6, 98.4) | 31.2 (-5.8, 55.3) | 94.0 (76.0, 98.5) | 37.2 (2.2, 59.6) |

CF: counterfactual; PY: person-years; CAB-LA: Cabotegravir; FTC/TDF: emtricitabine/tenofovir disoproxil fumarate; STI: sexually transmitted infection; HR: Hazard Ratio: CI: confidence interval

# **Table S6. DBS Biomarker measuring PrEP use in HPTN 084, AMP-women’s and HVTN702 trials**

|  | HPTN 084 FTC/TDF arm: Proportion with quantifiable TFV-DP in DBS | HPTN 084 FTC/TDF arm: Proportion with TFV-DP in DBS  >700 fmol/punch | AMP-women:  (Weighted) Proportion with quantifiable TFV-DP in DBS | AMP-women: (Weighted) Proportion with TFV-DP in DBS  >700 fmol/punch | HVTN 702:  (Weighted) Proportion with quantifiable TFV-DP in DBS | HVTN 702:  (Weighted) Proportion with quantifiable TFV-DP in DBS |
| --- | --- | --- | --- | --- | --- | --- |
| Five-country comparison in Women, age 18-40 | 70.5% | 21.0% | 4.3% | 0.5% | NA | NA |
| Three-country comparison in Women, age 18-35 | 68.6% | 20.6% | NA | NA | NA | NA |
| South Africa only,  age 18-35 | 70.6% | 20.1% | NA | NA | 2.8% | 0.2% |

DBS: dried blood spot; PrEP: pre-exposure prophylaxis: TFV-DP: tenofovir diphosphate

# **Methods: Direct standardization method for counterfactual incidence estimation**

**Step 1: Construct incidence estimates**

Let $i$ be the index of the set of *S* strata or categories (e.g., country x age category x baseline STI), $i=1,\ldots,S$. Then, the counterfactual incidence corresponding to S, for the external reference study is given by

$$CF\left( S \right)= {\sum_{i} m_{i}I_{i}}/{\sum_{i} m_{i}}$$

where

$m_{i}$= total number of person-years in category $i$ of the active product group in HPTN 084

$I_{i}$ = incidence in unit i in the external reference study

= $\frac{y_{i}}{n_{i}}$

where $y_{i}$ and $n_{i}$ are number of incident infections and person-years from the external reference study category $i$.

Then

$$\text{Var}\left( CF\left( S \right) \right)={\sum_{i} m_{i}^{2}{\text{Var}(I}_{i})}/{\left( \sum_{i} m_{i} \right)^{2}}$$

where

$Var\left( I_{i} \right)= {y_{i}}/{n_{i}^{2}}$

Using the delta method, the variance Is: $\mathrm{Var}\left( \log\left( \mathrm{CF}\left( S \right) \right) \right)={\mathrm{Var}\left( \mathrm{CF}\left( S \right) \right)}/{\left( \mathrm{CF}\left( S \right) \right)^{2}}$ .

This approach is equivalent to using a survey sampling Poisson regression model on the individual-level data in the external cohort with sampling weights:

$$w_{i}= \frac{m_{i}}{n_{i}}\times\frac{\sum_{i} n_{i}}{\sum_{i} m_{i}}$$

**Step 2: Construct counterfactual efficacy estimate**

The counterfactual efficacy estimate assumes that incidence rates estimated from the external study are representative of HIV risk within each strata category in the absence of study provision of active PrEP product, and the data from different studies are independent.

Compute the observed (unweighted) incidence for the active product group for *S*

$$O\left( S \right)= {\sum_{i} z_{i}}/{\sum_{i} m_{i}}$$

where

$z_{i}$ = seroconversions in category i of active product group

$$var\left( O\left( S \right) \right)= {\sum_{i} z_{i}}/{\left( \sum_{i} m_{i} \right)^{2}}$$

The counterfactual relative risk ratio is estimated for S:

$$RR\left( S \right)=\frac{O\left( S \right)}{CF\left( S \right)}= \frac{\sum_{i} z_{i}}{\sum_{i} m_{i}I_{i}}$$

$$Var(\log RR(S))= \frac{Var O\left( S \right)}{({O(S))}^{2}}+\frac{Var CF\left( S \right)}{{CF\left( S \right)}^{2}}= \frac{1}{\sum_{i} z_{i}}+\frac{\sum_{i} m_{i}^{2}\frac{y_{i}}{n_{i}^{2}}}{\left( \sum_{i} m_{i}\frac{y_{i}}{n_{i}} \right)^{2}}$$

**Step 3: Comparison of baseline demographics**

Since the person-year distributions of the external study and the active control study are matched, we compare the baseline demographics weighted by person-years. Specifically, the summary for the baseline demographics for the active-control study is generated by weighting each subject by the follow-up time. The summary for the baseline demographics for the matched external study is generated by weighting each subject by the product of her follow-up time and the corresponding subgroup weight $w_{i}$ given in Step 1.

# **R code for the counterfactual analysis**

# Calculate counterfactual placebo incidence

# mi: PY in current trial; yi: PY in placebo trial; ni event in placebo trial

# CF: incidence estimate; varCF: variance estimate; CICF: confidence interval; wi: individual weight

cal_CF <- function(mi,yi,ni){

Ii = yi/ni; varIi = yi / ni^2

CF = sum(mi*Ii)/sum(mi)

varCF = sum(mi^2 * varIi) / sum(mi)^2

CICF = c(CF*exp(qnorm(0.025)*sqrt(varCF)/CF),CF*exp(qnorm(0.975)*sqrt(varCF)/CF))

return(list(CF = CF, varCF = varCF, CICF = CICF, wi = mi*sum(ni)/sum(mi)/ni))

}

# Calculate observed incidence

# mi: PY in current trial; zi event in current trial

# O: incidence estimate; varO: variance estimate; CIO: confidence interval

cal_O <- function(zi,mi){

O = sum(zi)/sum(mi)

varO = sum(zi)/(sum(mi)^2)

CIO = c(O*exp(qnorm(0.025)*sqrt(varO)/O),O*exp(qnorm(0.975)*sqrt(varO)/O))

return(list(O = O, varO = varO, CIO = CIO))

}

# Calculate relative risk

# res_CF: counterfactual placebo estimate from cal_CF()

# res_O: observed incidence estimate from cal_O()

# RR: estimated relative risk; varRR: variance estimate; CIRR: confidence interval

cal_RR <- function(res_CF,res_O){

RR = res_O$O / res_CF$CF

varRR = RR^2 * (res_O$varO/(res_O$O^2) + res_CF$varCF/(res_CF$CF^2))

CIRR = c(RR*exp(qnorm(0.025)*sqrt(varRR)/RR),RR*exp(qnorm(0.975)*sqrt(varRR)/RR))

return(list(RR = RR, varRR = varRR, CIRR = CIRR))

}
